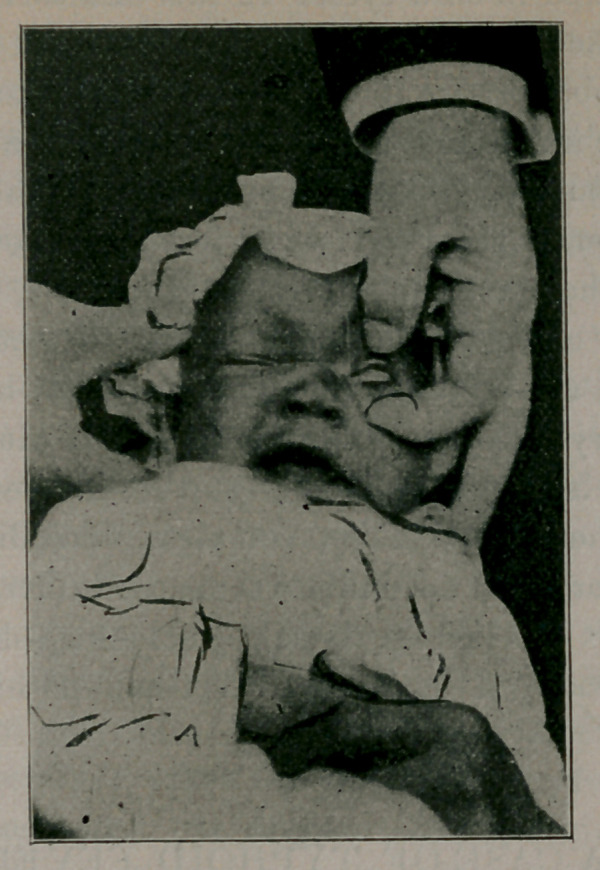# Ankyloblepharon

**Published:** 1905-10

**Authors:** Wm. Lewis Bullard

**Affiliations:** Columbus, Ga.


### ANKYLOBLEPHARON.

By Wm. LEWIS BULLARD, M. D., Columbus, Ga.

Two weeks ago this rare case was brought to me—a negro child male, six months old. On inquiry I learned that this case was not congenital, but acquired. When born, and for three weeks afterwards the eyes were normal. About this time it had "sore eyes," possibly croupous conjunctivitis or may be blepharitis marginalis. It was not seen by a physician, though various remedies and receipts were advised by friends and acquaintances, most of which were used, including an old favorite "chamberlye"—fresh from the babe.

Ankyloblepharon may be congenital, but is more often caused from traumatic lesions, especially burns, though it may be the result of an ulceration of the mucous membrane and extending to the ciliary margin. It is not often that we find the palpebral margins completely adhered in both eyes. In this case such was so, save a small opening the size of a pin head on the nasal side. With a pair of small probe scissors it was an easy matter to introduce and divide the palpebral adhesions, to whose edges an application of flexible collodium was adjusted to prevent readhesion. The babe looked wondrously surprised when for the second time (when born and after the operation) during its short life, this wonderful world had been opened unto the "windows of the soul." Cases like this (which are very seldom) the treatment is easy and the result satisfactory. Not so when the trouble is caused from burns. Here treatment is very questionable and results never satisfactory, especially so when adhesions are complete, including conjunctiva and ball; therefore, I'll not inflict the many readers of this journal with its technic. I inclose kodak photo-snap-shot and report the case on account of its rarity, "this and nothing more."

## Figures and Tables

**Figure f1:**